# Godey’s Lady’s Book

**Published:** 1856-06

**Authors:** 


### GODEY'S LADY'S BOOK.

WELL done! A monthly periodical which can be published over a quarter of a century and never allow a profanity to pollute its pages, makes another step forward, which is worthy of all praise, and which doubly shames nine-tenths of the religious newspapers of the day, in asserting in the number for the present month, from Philadelphia:--

"We were offered a few days since a very handsome sum of money if we would allow a pamphlet containing a notice of quack medicines to be directed to each of our subscribers. We declined, as we did not choose to make them pay postage on an article which could be of no possible use to them." How is it that Godey's Lady's Book can make its proprietor independent without prostituting it to profanity, uncourteousness and quackery, and yet the religious press, in order to "*make it pay,*" will publish without stint, some of the most palpable and unblushing falsehoods, in reference to medicine and systems of medicine, to which they would not trust the life of a valuable horse, let alone their own lives.

